# Diet Advice for Crohn’s Disease: FODMAP and Beyond

**DOI:** 10.3390/nu12123751

**Published:** 2020-12-06

**Authors:** Stefan L. Popa, Cristina Pop, Dan L. Dumitrascu

**Affiliations:** 1 2nd Medical Department, Faculty of Medicine, Iuliu Hatieganu University of Medicine and Pharmacy, 400006 Cluj-Napoca, Romania; popa.stefan@umfcluj.ro (S.L.P.); ddumitrascu@umfcluj.ro (D.L.D.); 2 Department of Pharmacology, Physiology, and Pathophysiology, Faculty of Pharmacy, Iuliu Hatieganu University of Medicine and Pharmacy, 400349 Cluj-Napoca, Romania

**Keywords:** Crohn’s disease, low FODMAP diet, inflammatory bowel disease (IBD), diet, nutrition, therapy, vegan diet, vegetarian diet, Mediterranean diet

## Abstract

Crohn’s disease (CD) is a chronic, progressive, and destructive granulomatous inflammatory bowel disorder that can involve any part of the gastrointestinal tract. It has been presumed that different types of diet might improve gastrointestinal symptoms in CD patients. The aim of this review was to clarify the efficiency and indications of a low-“fermentable oligo-, di-, mono-saccharides and polyols” (FODMAP) diet (LFD) in CD and to further analyze the available data on other types of diets. PubMed, Cochrane Library, EMBASE and WILEY databases were screened for relevant publications regarding the effect of FODMAP diets on CD. Our search identified 12 articles analyzing the effect of an LFD in CD, 5 articles analyzing the effect of a Mediterranean diet (MD), 2 articles analyzing the effect of a vegetarian diet (VD), and 2 articles analyzing the effect of a low-lactose diet (LLD). The majority of the studies included in this review show the significant efficiency of the LFD in CD patients. We found significant evidence demonstrating that the LFD has a favorable impact on gastrointestinal symptoms in CD patients. Notwithstanding the evidence, it remains to be established if an LFD is more efficient than other types of diets in the short term and especially in the long term.

## 1. Introduction

Crohn’s disease (CD) is a chronic, progressive, and destructive granulomatous inflammatory bowel disorder that can involve any part of the gastrointestinal tract from mouth to anus, predominantly the terminal ileum, ileocaecal region, colon, and perianal region [1,2,3]. The digestive symptomatology includes bloody mucopurulent diarrhea, abdominal pain, nausea, emesis, weight loss, perineal pain, and urgency to defecate while arthritis, anorexia, uveitis, and skin rash represent the main extra-intestinal manifestations. CD patients frequently experience periods of symptomatic relapse and remission [1,2,3]. While no gold standard for the diagnosis of Crohn’s disease exists, the diagnosis is made by a combination of clinical, endoscopic, histological, imagistic, and biochemical criteria. World Health Organization diagnostic criteria for CD include: discontinuous or segmental lesions, as well as a cobblestone appearance or longitudinal ulcer, noted on radiologic studies, endoscopy, and resected specimens; transmural inflammation, as evidenced by clinical evaluation, radiologic studies, biopsy findings, and resected specimens; noncaseating granulomas, as revealed on biopsy samples and resected specimens; fissures and fistulas, as evidenced by clinical evaluation, radiologic studies, and resected specimens; perianal disorders upon clinical evaluation [4,5,6,7,8]. Nevertheless, for 15% of patients initially diagnosed with CD, the diagnosis changes to ulcerative colitis (UC) during the first year [5,6,7].

A low-“fermentable oligo-, di-, mono-saccharides and polyols” (FODMAP) diet is often used in irritable bowel syndrome (IBS) patients because symptoms of bloating, cramping, and diarrhea may be reduced by a diet that will limit foods high in fructose, lactose, fructans, galactans, and polyols [9]. FODMAPs are osmotic short-chain carbohydrates that are not completely absorbed in the small intestine, absorb water and are fermented by the bacteria in the distal small and proximal large intestine. The result of this cycle is the production of gas, which could partially explain the bloating and flatulence [9,10,11]. The daily intake of FODMAPs in a normal diet ranges from 15 g to 30 g per day. If the low-FODMAP diet (LFD) was considered an “avoidance diet” in the past, the present approach is also considering it as a diagnostic tool test in several gastrointestinal and non-gastrointestinal disorders [9,10,11]. FODMAPs have considerable osmotic properties, compelling water into the gastrointestinal lumen. Inside the colon, FODMAPs are easily and quickly metabolized by gut microbiota followed by the process of fermentation, and the result is an increased quantity of gas, which leads to abdominal distention and diffuse abdominal pain [12,13,14,15].

The Mediterranean diet (MD) is characterized by a high intake of plant-based foods, unrefined cereals, fruit, vegetables, legumes, olive oil as the main source of fat, moderate to high consumption of fish, moderate consumption of dairy products (mostly as cheese and yogurt) and low consumption of non-fish meat products [16,17,18]. Although all the countries from the Mediterranean region have different diets, influenced by geographical, economic, historical, or religious parameters, it is considered that these subtypes are variations of the same MD diet with insignificant differences [16,17,18]. MD is associated with low risk of late-onset CD and because it frequently presents high adherence among patients, it has been demonstrated to improve quality of life and reduce intestinal inflammation.

The low-lactose diet (LLD) is usually used as a treatment for patients with a long history of bloating, gas, diffuse abdominal pain, nausea, and diarrhea, resulting from incomplete digestion of lactose and refractory to different types of therapy [19,20,21,22,23].

In spite of the remarkable progress in the development of new pharmacological agents, which show considerable efficiency, there is no medication that can treat all patients efficiently, probably because of the genetic and phenotypic heterogeneity.

The aim of the present narrative review was to clarify the efficiency and indications of an LFD in CD and to further analyze available data on other types of diets.

## 2. Materials and Methods

**Figure 1:**
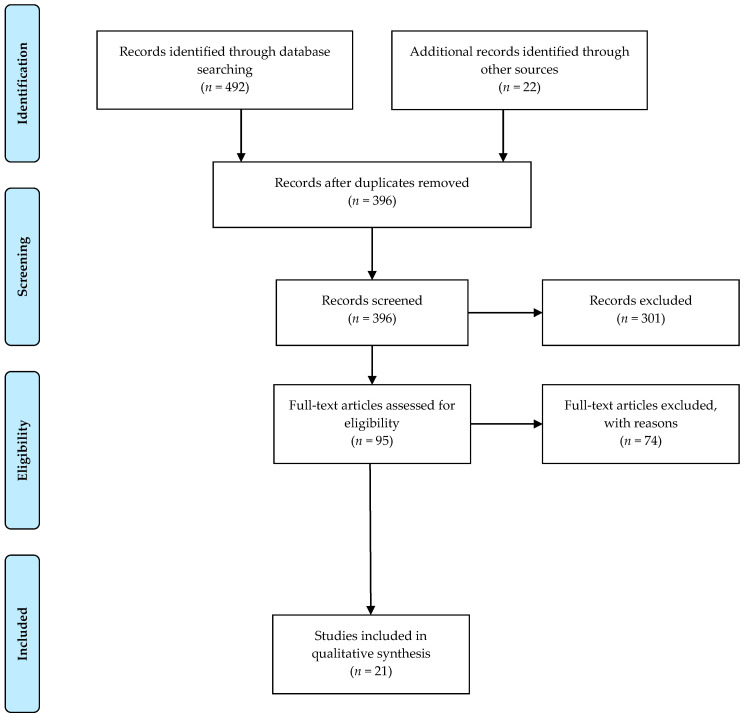
Flow diagram for study selection.

PubMed, Cochrane Library, EMBASE, and WILEY databases were screened for relevant publications about the effect of the FODMAP enteral diet on CD. The search terms included: (Crohn’s Disease OR Crohn OR Crohn Disease OR Inflammatory Bowel Disease OR IBD) AND (FODMAP OR Diet OR fermentable oligosaccharides disaccharides monosaccharides polyols OR Mediterranean diet OR vegetarian diet OR ovo-lacto vegetarian diet OR semi-vegetarian diet OR low-FODMAP diet OR low-lactose diet). The exclusion criteria were: studies written in languages other than English, case reports, pediatric studies, abstracts, conference presentations, letters to the editor, and editorials (Figure 1).

## 3. Results

### 3.1. Low-FODMAP Diet

Our search identified 12 articles analyzing the effect of an LFD in CD (Table S1). There are several trials looking at the effects of dietary interventions in CD. Gershon et al. showed that an LFD overstimulates the Meissner’s plexus by vegetative neuroenteric sensory transmission, leading to an increased intestinal secretion and increased gastrointestinal motility and a shorter transit time resulting in lower inflammation [14]. In order to verify the anti-inflammatory effect of an LFD, Zhou, et al. hypothesized that a high-FODMAP diet (HFM) increases the intestinal inflammatory activity, barrier dysfunctions, and visceral hypersensitivity [24]. Experiments on rats were performed to test this hypothesis. Rats were fed with an HFM and mucosal inflammatory status and the gene expression of inflammatory cytokines were measured in colonic tissues [24]. The results showed that the level of IL-1β, IL-6, IL-17, TNF-α, and IFN-γ mRNA increased significantly in the group of rats fed with HFM, compared with standard fed rats (*p* < 0.05) [24]. Furthermore, the authors examined the lamina propria of the colon from rats fed with HFM compared with rats fed with standard food. The results showed an increased number of mononuclear cells, neutrophils, eosinophils, and mast cells in the HFM group, demonstrating the presence of low-grade mucosal inflammation after an HFM diet [24]. A study performed by Hustoft et al. on 20 IBS patients showed a decreased serum level of IL-6, IL-8, and n-butyric acid, after following an LFD for a period of 21 days, demonstrating the anti-inflammatory effect of an LFD [25]. Other studies have shown the LFD mechanisms and effects on symptomatology and cytokine levels, but the vast majority of studies were performed on IBS patients and fewer on IBD or CD patients alone [25,26,27,28,29,30,31,32,33].

A pilot study performed by Gearry et al. on 52 CD consecutive patients and 20 UC patients analyzed the effect of an LFD on gut symptoms [34]. The results showed that abdominal pain, bloating and flatulence improved in CD patients and the number of diarrheic stools decreased, but the LFD had no effect on constipation [34]. Furthermore, the efficiency of the LFD was associated with adherence to an LFD (*p* = 0.033) and inefficacy with non-adherence to an LFD (*p* = 0.013) [34]. A randomized, controlled trial conducted by Cox et al. made a complex investigation into the effects of an LFD in CD and UC quiescent patients, evaluating gut symptomatology, markers of inflammation and the intestinal microbiome [35]. Patients were divided into two study groups, the LFD group and the control diet group, and gastrointestinal symptomatology and quality of life were measured using validated questionnaires. Blood samples and fecal microbiome composition and function were assessed at baseline and at the end of the study using shotgun metagenomic sequencing and phenotypes. T cells were analyzed from the blood samples using flow cytometry [35]. The results showed that more than a half of patients reported amelioration of gastrointestinal symptoms after adherence to an LFD (14/27, 52%) compared to 16% of the control diet group (*p* = 0.007). The results obtained by validated questionnaires investigating the quality of life (QoL) showed that patients from the group had higher health-related QoL scores (81.9 ± 1.2) than patients from the control diet group (78.3 ± 1.2, *p* = 0.042) [35]. *Bifidobacterium adolescentis*, *Bifidobacterium longum*, and *Faecalibacterium prausnitzii* were present in a lower quantity in the group than in the control diet group [35]. On the contrary, with the trial hypothesis, serum inflammation markers did not differ between the study groups, nor did the microbiome diversity [35]. Because no adverse effects appeared during the 4 weeks of the trial, the authors concluded that an LFD is safe and efficient for managing persistent gastrointestinal symptomatology in IBD patients [35].

A randomized, controlled cross-over trial conducted by Halmos et al. analyzed the effects of an LFD on markers of colonic health of eight CD patients [36]. The patients were adherent to an LFD for a period of 21 days, and gut symptoms were recorded daily. During a period of 5 days, fecal samples were collected at the end of each diet and analyzed for pH, short-chain fatty acids (SCFA), calprotectin, and bacterial abundance [36]. The results showed that after an LFD, SCFA, pH, and total bacterial abundance were the same, but relative abundance was higher for butyrate-producing *Clostridium* cluster XIVa (*p* = 0.008) and mucus-associated *Akkermansia muciniphila* (*p* = 0.016), and lower for *Ruminococcus torques* (*p* = 0.034) [36]. Furthermore, LFD had no effects on calprotectin levels [36]. The main limitation of this trial is the small number of CD patients included in the study.

It has been previously demonstrated that the standard Egyptian daily diet contains a high amount of FODMAP [37]. A study on 100 Egyptian CD patients in remission performed by Elhusseiny et al. analyzed the impact of an LFD on the functional gastrointestinal disorders (FGID) symptoms and the quality of life (QoL) [37]. The results showed that the improvement was 38.45 ± 21.56% regarding the mean score of FGID. The QoL was significantly improved, especially in women (90% versus to 49.4% in males), who had a better quality of life [37]. Although 67% of CD patients were adherent to the LFD (18.16 ± 6.85), the rest of the patients were not adherent because the LFD was too expensive [37].

A randomized, double-blinded, placebo-controlled, re-challenge trial conducted by Cox et al. analyzed 29 patients with IBD (12 CD patients and 17 UC patients) fulfilling criteria for IBS, functional bloating, or functional diarrhea [38]. During the study, IBD patients were allocated to a series of 3-day fermentable carbohydrate diets in a random order (fructan, 12 g/d; galacto-oligosaccharides (GOS) 6 g/d; sorbitol, 6 g/d; and glucose placebo, 12 g/d) [38]. The results showed that a considerable but small number of patients reported an improvement in functional gastrointestinal symptomatology after the fructan diet (18/29, 62.1%) compared with the glucose diet (26/29, 89.7%) (*p* = 0.033) [38]. Furthermore, the results showed a greater severity of pain (1.1 vs. 0.5, *p* = 0.004), bloating (1.3 vs. 0.6, *p* = 0.002), flatulence (1.5 vs. 0.7, *p* = 0.004), and faecal urgency (0.9 vs. 0.4, *p* = 0.014) after a fructan diet compared with a glucose diet. The authors concluded that fructans and not GOS or sorbitol exacerbated functional gastrointestinal symptomatology in IBD patients [38].

A randomized, double-blind, placebo-controlled trial conducted by Benjamin et al. with predefined clinical microbiological and immunological endpoints analyzed the impact of a diet with fructo-oligosaccharides (FOS) in 103 CD patients [39]. The trial protocol divided CD patients into two study groups: placebo group (*n* = 49) and FOS group (*n* = 54). The results showed that patients from the FOS group presented a decreased level of interleukin 6 (IL-6) and an increased level of immunoregulatory dendritic cell staining of IL-10 (*p* < 0.05) [39]. The study found no correlation between the level of IL-12p40 and FOS diet in CD patients. Furthermore, no significant differences in the fecal concentration of bifidobacteria and *F. prausnitzii* were found between the study groups at baseline or after the 4-week FOS dietary intervention [39].

A study performed by Prince et al. on 88 patients with IBD aimed to investigate the efficiency of an LFD on 88 IBD patients with coexisting FGID symptomatology [40]. The study protocol used the Gastrointestinal Symptoms Rating Scale to assess gastrointestinal symptoms and the Bristol Stool Form Scale for the stool analysis [40]. Gastrointestinal symptoms were analyzed at baseline and at a minimum of six weeks of follow-up, and the results showed that there was a significant increase in patients with IBD declaring a relief of gastrointestinal symptoms between the baseline (14/88, 16%) and LFD (69/88, 78%; *p* < 0.001) [40]. The efficiency of an LFD was also demonstrated by an improvement in stool consistency and frequency, which included an increase in “normal” stool form (*p* = 0.002) and “normal” stool frequency (*p* < 0.001) [40].

A cross-sectional study performed by de Castro et al. compared the effects of three different diets on 60 CD patients with CD [41]. The study protocol provided a validated food frequency questionnaire (FFQ) used to collect dietary intake data and consisted of 76 food items further classified into 22 food groups. The results of the study showed that an LFD was associated with symptoms, gender and duration of disease, a diet high in snacks and processed foods was positively associated with CD duration and negatively associated with age, while a diet high in fruits, vegetables and eggs was positively associated with physical activity and negatively associated with smoking and BMI [41]. Associations between eating patterns and the stage of the disease were not confirmed by this study [41].

A study performed by Linsday et al. analyzed the effect of the administration of FOS on CD activity, bifidobacteria fecal concentrations, and the function of mucosal dendritic cells [42]. The study protocol included 10 CD patients. CD activity was assessed using the Harvey–Bradshaw index. Furthermore, flow cytometry from rectal biopsies was performed in order to analyze dendritic cell IL-10 and Toll-like receptor (TLR) expression [42]. Fecal and mucosal bifidobacteria concentrations were determined by fluorescence in situ hybridization [42]. The results showed that administration of 15 g of FOS during a study period of three weeks decreased CD activity because dendritic cells expressing TLR2 and TLR4 increased from 1.7 (1.7)% to 36.8 (15.9)% (*p* = 0.08) and from 3.6 (3.6)% to 75.4 (3.4)% (*p* < 0.001); IL-10 positive dendritic cells increased from 30 (12)% to 53 (10)% (*p* = 0.06) and faecal bifidobacteria concentration increased from 8.8 (0.9) log (10) to 9.4 (0.9) log(10) cells/g dry faeces (*p* < 0.001) [42].

### 3.2. Mediterranean Diet

Our search identified five articles analyzing the effect of MD in CD (Table S1). A prospective cohort study conducted by Khalil et al. analyzed the risk of late onset Crohn’s disease in 83,147 subjects with ages ranging from 45 to 79 years enrolled in the Cohort of Swedish Men and the Swedish Mammography Cohort [16]. Subjects were included in the study in 1997, and the results were analyzed in December 2017. After an average follow-up of 17 years, 164 cases of CD were confirmed. The conclusion of the study was that greater adherence to an MD was associated with a significantly lower risk of late onset CD [16]. Furthermore, age, sex, education level, body mass index and smoking did not modify these associations (all P interaction > 0.30) [16]. Because QoL is impaired in CD patients, Papada et al. analyzed 86 CD patients. The protocol of the study included: medical history, disease activity, dietary intake, habitual Mediterranean diet (MedDiet) score, anthropometric measurements, and Inflammatory Bowel Disease Questionnaire (IBDQ). All of these protocols were recorded, as well as blood samples for quantification of biochemical and inflammatory indices [17]. Patients with inactive CD had greater adherence to MD. The results showed that MedDiet score was positively correlated with the IBDQ score (*p* = 0.008) and negatively with disease activity (*p* < 0.001), demonstrating that adherence to MD is associated with an improved quality of life in CD patients and reduced disease activity [17]. A study performed by Chicco et al. analyzed the nutritional state, clinical disease activity, quality of life (QoL), and liver steatosis in both CD and UC patients following an MD for a period of 6 months. In order to show the effects of MD, body mass index (BMI), body tissue composition, liver steatosis and function, serum lipid profile and inflammatory biomarkers (C-reactive protein and fecal calprotectin) were collected at baseline and after 6 months [17]. The results showed that MD had a significant effect on improving QoL and reducing the inflammatory state, but no effect on serum lipid profile or liver function [17]. Because patients with CD frequently present nutritional deficiency, Taylor et al. analyzed dietary patterns of CD patients by comparing the micronutrient intakes of CD patients with a representative sample of individuals and further analyzing the macro- and micro-nutrient intakes in CD patients following an MD [43]. Although MD presented anti-inflammatory properties in CD patients, patients with CD reported food choices that promote a more restricted nutrient intake. Thus, the authors of the study recommend that all CD patients should be screened for nutrient deficiencies and choosing a specific diet should be a tailored process involving more clinical, biological, imagistic, endoscopic, and histologic parameters [43]. Using complex transcriptomics technologies, Marlow et al. analyzed the effect of MD on a population with CD [44]. After a period of 6 weeks on an MD, values for inflammation biomarkers such as C-reactive protein and micronuclei numbers significantly improved [44]. The results showed a positive effect and significant changes in overall gene expression, with 3551 genes presenting altered expression as a result of the MD [44]. Furthermore, MD normalized the microbiota, demonstrating the effects of an MD on the gut microbiome.

### 3.3. Low-Lactose Diet

Our search identified two articles analyzing the effect of an LLD on CD (Table S1). A study performed by Gudmand-Hoyer et al. on 71 CD patients and 85 UC patients of investigated the lactose malabsorption incidence in IBD patients using lactose tolerance tests and determinations in the small intestinal mucosa of disaccharidase [19]. The results of the study showed that four CD patients (6%) and eight UC patients (9%) had lactose malabsorption and the control group displayed a similar incidence [19]. The authors concluded that lactose malabsorption was not particularly common in IBD, and its pathogenesis is unrelated to the inflammatory disease. Furthermore, nine CD patients and 21 UC patients followed an LLD, and a beneficial effect was noticed in three CD patients and five UC patients [19]. A case–control study performed by Capristo et al. analyzed the effects of a standard polymeric diet or a vegetable protein-rich and lactose-free diet in inactive CD patients [20]. The study protocol provided that all CD patients were clinically examined and laboratory tests were also performed [20]. Substrate oxidation rates were measured by indirect calorimetry, and body composition was analyzed using isotopic dilution and resting metabolic rate [20]. The results showed that a polymeric enteral diet rich in vegetable protein and not containing milk protein, eaten at home, without a nasogastric tube, improved body composition and significantly improved the nutritional status in CD patients [20].

## 4. Discussion

Studies show that dietary intervention in CD may have a positive impact regarding a patient’s quality of life, symptom reporting, inflammatory markers and overall disease progression, while, for the vegetarian (and derivates such as ovo-lacto vegetarian and semi-vegetarian diets) and low-lactose diets, the evidence is not yet compelling [45,46]. For Mediterranean and FODMAP diets, there are numerous studies presenting the benefits of these interventions [15,47,48,49,50].

In the past five decades, the overall FODMAP intake has considerably increased in the Western world because of the use of high-fructose corn syrup in almost all processed foods, ranging from juice, bread, yogurt, frozen junk food, salad dressings and even granola bars, macaroni, cheese and canned fruit [15,47,48,49,50]. Because FODMAPs distend the small bowel, as a consequence of increased intraluminal secretion of water and increased gas production, the hypothesis that an LFD could prevent FGID symptoms was widely tested in IBS patients.

Nevertheless, in the last decade, the same hypothesis was tested not only in FGID but also in organic gastrointestinal disorders. Several systematic reviews and meta-analyses investigated the efficiency of an LFD on the clinical and biological outcomes of CD patients. The majority of those studies concluded that an LFD had the following effects: amelioration of gastrointestinal symptoms, the number of diarrheic stools decreased, decreasing of the inflammatory markers, and improved scores in QoL questionnaires indicating an improvement of general well-being [13,51,52,53,54,55]. Because current guidelines have insufficient recommendations for diet indications in CD, international cooperation is needed in order to overcome the present limitations represented by the lack of comparative studies analyzing different types of diets and the small number of patients included in the available studies. More studies are required to increase the level of evidence for dietary intervention in CD.

The main limitations of our study were the insufficient number of studies about the efficiency of VD and LLD (only two articles for each diet) and the small number of patients included in the majority of studies. Another limitation was the fact that a considerable number of studies analyzed the effect of different types of diet in both CD and UC patients. Our search did not find sufficient data about the efficiency of other types of diet in CD patients (vegan diet, low fiber diet, gluten-free diet, ketogenic diet), and the available studies include a limited number of patients with inconclusive results [21,22,23]. Although there considerable progress has been made with regard to enteral nutrition in CD, containing macro- and micro-nutrients [56], we did not analyze this subject because our search included only data about oral nutrition. Other studies provide evidence of the efficiency of the different types of diets in CD [33,57,58,59,60,61,62,63,64,65,66], but no official guidelines regarding nutrition in CD are available. Even though the majority of studies have demonstrated the efficiency of an LFD in CD patients [13,51,52,53,54,55], the fact that an LFD is highly restrictive, expensive and difficult to follow requires the active support of a dietician. Only a small percentage of the initially excluded foods are excluded until the end of the study, and this must be mentioned as a major limitation of those studies, this being the source of the heterogeneity among the overall results.

## 5. Conclusions

We found significant evidence demonstrating that an LFD has a favorable impact on gastrointestinal symptoms in CD patients. Notwithstanding the evidence, it remains to be established whether an LFD is more efficient than other types of diets in the short term and especially in the long term.

## Supplementary Materials

The following are available online at https://www.mdpi.com/2072-6643/12/12/3751/s1.
