# Supplementary material for: Diet Advice for Crohn’s Disease: FODMAP and Beyond
Source: Nutrients. 2020 Dec 6;12(12):3751. doi: 10.3390/nu12123751 (PMC7762196; doi:10.3390/nu12123751)
Supplement: Supplementary file 1 [file nutrients-12-03751-s001.pdf]

Table S1. Studies Assessing the Outcomes after LFD and other types of diet in CD patients.

First Author	Publication Year	Country	Study Design	Total Patients	Mean Age (years) (mean ± SD) /(Range)	Diagnosis	Sex (%Male)	Main Findings
Gearry RB et al. [34]	2009	Australia	Retrospective questionnaire-based study	72	48 (18–72)	52—Crohn's disease 20—ulcerative colitis	45.83%	Reduction in FODMAP intake was an efficacious strategy for patients with IBD.
Cox SR et al. [35]	2020	UK	Multicenter, randomized, parallel, single-blinded, placebo-controlled trial	52	36 ± 12	Quiescent Crohn's disease or ulcerative colitis and persistent gut symptoms Rome III criteria	44.23%	The low-FODMAP diet reduced fecal microbial abundance without significant effect on inflammation markers. Patients with quiescent IBD can benefit from a 4-week diet low in FODMAPs.
Halmos EP et al. [36]	2016	Australia	Randomized, controlled, blinded, cross-over trial	8	35 (29–41)	Clinically quiescent Crohn's disease Harvey-Bradshaw Index of ≤5	25%	Altering dietary FODMAP intake is associated with changes similar to a prebiotic effect on the gut.
Elhusseiny MH et al. [37]	2018	Egypt	Cross-sectional interventional	100	28.56 ± 7.0	Crohn's disease in remission C-reactive protein (CRP) (<3 mg/L), fecal calprotectin (<100 µg/g), Crohn's disease activity index (CDAI) below 150 points, and normal findings by computed tomography and endoscopic reports	42%	The low-FODMAP diet was effective in improving the quality of life of CD patients with FGID.
Cox SR et al. [38]	2017	UK	Randomized, double-blind, placebo-controlled, cross-over, re-challenge trial	29	39 ± 19.5	IBD: Crohn's disease (12), ulcerative colitis (17)	34.4%	At high doses, fructans exacerbated FGS compared to GOS or sorbitol, in quiescent IBD patients.
Benjamin JL et al. [39]	2011	UK	Randomized, double-blind, placebo-controlled trial	103	FOS 40 ± 14.8 Placebo 39 ± 13.7	Crohn's disease	FOS 33% Placebo 45%	In patients with active Crohn's disease FOS presented with no clinical benefit.
Prince AC et al. [40]	2016	UK	Cross-sectional interventional	88	40 ± 13	IBD: Crohn's disease (39), ulcerative colitis (38) IBD-u (11)	30%	The low-FODMAP diet was effective in improving FGS in IBD.

de Castro MM et al. [41]	2020	Brazil	Cross-sectional interventional	60	18–60	Crohn's disease—endoscopic, radiological, and histological criteria	51.7%	Three dietary patterns were identified: "Traditional + FODMAP", "Fitness style" and "Snacks and processed foods".
Lindsay JO et al. [42]	2006	UK	Open-label trial	10	40 (29–56)	Moderately active colonic or ileocolonic Crohn's disease Harvey–Bradshaw Index (HBI) >5	80%	FOS supplementation increases fecal bifidobacteria concentrations and decreases Crohn's disease activity.
Khalili H et al. [16]	2020	Sweden	Population-based prospective cohort study	83147	45–79	Crohn's disease, ulcerative colitis	55.21%	Significantly lower risk of late onset CD was obtained with greater adherence to MD.
Papada E et al. [17]	2020	Greece	Cross-sectional	86	Active disease 41.5 ± 16.5 Inactive disease 37.4 ± 11.5	Crohn's disease —relapse (5 ≤ Harvey–Bradshaw Index ≤ 14) —remission (Harvey–Bradshaw Index ≤ 4)	64.9%	Higher quality of life in CD patients is achieved with adherence to MD.
Chicco F et al. [18]	2020	Italy	Prospective, interventional study	142	Crohn's disease 48 (34.8–57.5) Ulcerative colitis 52 (44.5–61.3)	Crohn's disease, ulcerative colitis	53.5%	MD was associated with a spontaneous improvement of disease activity in IBD.
Taylor L et al. [43]	2018	Canada	Single center cross-sectional study	67	45	Crohn's disease clinical, radiological, and endoscopic criteria Harvey–Bradshaw Index (HBI) <5	50.74%	Patients with CD reported a more restrictednutrient intake. P-MDS is an effective tool to identify pro-inflammatory dietary patterns.
Marlow G et al. [44]	2013	New Zealand	Cross-sectional	8	45.4 (31–60)	Crohn's disease	25%	Mediterranean-inspired diet reduced markers of inflammation and normalized the microbiota of CD patients.
Schreiner P et al. [45]	2019	Switzerland	Population-based cohort study	1254	Not vegetarian 29.6 (21.7–39.6), Vegetarian 28.1 (20.6–35.8)	Crohn's disease, ulcerative colitis	52.47%	Patients on a VD or GFD had significantly lower psychological well-being.
Chiba M et al. [46]	2010	Japan	Prospective clinical trial	22	26.5 (19–77)	Crohn's disease	63.63%	SVD prevented relapse in CD.
Gudmand-Hoyer E et al. [19]	1970	Denmark	Cross-sectional	156	-	Crohn's disease, ulcerative colitis	41.6%	Lactose malabsorption is not common in IBD patients. If present, lactose intolerance is not related to IBD.

Capristo E et al. [20]	2000	Italy	Case–control study	40	26.6–42.4	Crohn's disease	50%	Polymeric enteral diet rich in vegetable protein, without milk protein, significantly improved body composition in CD patients.

IBS—irritable bowel syndrome, CD—Crohn's disease, HFM—high-FODMAP diet, LFM—low-FODMAP diet, LPS—lipopolysaccharides, FGID—functional gastrointestinal disease, GOS—galacto-oligosaccharides, FGS—functional gastrointestinal symptoms, IBD—inflammatory bowel disease, FOS—fructo-oligosaccharides, MD—Mediterranean diet, P-MDS—Mediterranean diet scores, VD—vegetarian diet, GFD—gluten-free diet, SVD—semi-vegetarian diet.
